# A Cross-Sectional Study of Empathy and Emotion Management: Key to a Work Environment for Humanized Care in Nursing

**DOI:** 10.3389/fpsyg.2020.00706

**Published:** 2020-05-13

**Authors:** María del Carmen Pérez-Fuentes, Ivan Herrera-Peco, María del Mar Molero Jurado, Nieves Fátima Oropesa Ruiz, Diego Ayuso-Murillo, José Jesús Gázquez Linares

**Affiliations:** ^1^Department of Psychology, Faculty of Psychology, University of Almería, Almería, Spain; ^2^Department of Psychology, Faculty of Psychology, Universidad Politécnica y Artística del Paraguay, Asunción, Paraguay; ^3^Nursing Department, Health Sciences Collegue, Alfonso X el Sabio University, Madrid, Spain; ^4^Colegio General de Enfermería, Madrid, Spain; ^5^Department of Psychology, Faculty of Psychology, Universidad Autónoma de Chile, Santiago, Chile

**Keywords:** care quality, emotional intelligence, cognitive empathy, nursing, humanization, healthcare

## Abstract

**Introduction:**

At the present time, technological advances have increased the technification of healthcare services, in which high priority is given to efficiency and results achieved, leading healthcare personnel to prioritize administrative and procedural aspects to the detriment of humanization of care and the work environment.

**Objective:**

This study was intended to continue progress in research on the work environment based on the humanization construct by analyzing the explanatory value of emotional intelligence and empathy in nursing personnel.

**Materials and Methods:**

The study was quantitative, observational, and cross-sectional. The sample was made up of 338 Spanish nurses with a mean age of 32.20 (SD = 7.54; range 22–56). The instruments employed for analysis were the Healthcare Professional Humanization Scale (HUMAS), Brief Emotional Intelligence Inventory for Adults, and Basic Empathy Scale (BES).

**Results:**

Mood and stress management—both emotional intelligence components—and cognitive empathy explained over half (51%) of the variability found in humanization of care in a sample of nurses. Furthermore, the mediation models proposed emphasized the mediating role of cognitive empathy in stress management and improvement in mood and its relationship to humanization.

**Conclusion:**

It is recommended that healthcare professionals reinforce their personal competencies in order to tend to the needs of their patients empathetically and improve emotional competencies for coping successfully with potentially stressful situations.

## Introduction

Although it may seem that there is a clear consensus with respect to the characteristics defining the work environment for humanization in healthcare, there is far from unanimous agreement. Studies to date on the value that certain psychological variables have for humanization of care have mainly dealt with empathy and communication skills (Beltrán, 2015; Espinosa et al., 2015; González-Hernández, 2015; Bautista et al., 2016; De la Fuente-Martos et al., 2018; Vásquez et al., 2018; Prado et al., 2019). The acquisition of these skills and abilities varies depending on the area of healthcare the nurses provide their services in Sharmay-Tsoory et al. (2004), Weiner and Auster (2007), Mortier et al. (2015), Howick et al. (2017), Wilkinson et al. (2017), Molero et al. (2018), Pérez-Fuentes et al. (2019b). In an attempt to systematize previous empirical evidence and, at the same time, contribute to the creation of a theoretical framework guiding intervention in the healthcare context in this direction, a multidisciplinary group of researchers have recently proposed the Healthcare Professional Humanization Scale (HUMAS) Healthcare Profession Humanization Model, based on the development of five personal competencies: Dispositional optimism, sociability, emotional understanding, self-efficacy, and affection (Pérez-Fuentes et al., 2019a, b). From this perspective, the humanization of healthcare is a professional competency, which can be acquired with practice. Professional practice in healthcare can be affected by dehumanization (Glebocka, 2019), which psychologically is due to patients losing their identity as individuals and no longer being perceived as active persons, but as being impaired, and the professional practice is performed mechanically, with lack of empathy, causing moral disengagement (Haque and Waytz, 2012). This depersonalization in providing healthcare has been associated with emotional exhaustion and stress (Murji et al., 2006; Parola et al., 2017; Molero et al., 2018; Busch et al., 2019), as well as environmental factors of nursing, such as staff ratios or patient care automation (Michelan and Spiri, 2018; Busch et al., 2019).

### Emotional Competencies in Healthcare

Emotional competencies are defined as “The knowledge, abilities, skills, and attitudes necessary to understand, express, and regulate appropriately emotional phenomena” (Bisquerra and Pérez, 2007, p. 69). They can be taught at school and in the family and must be practiced in the social and cultural context in which they take place. It has been demonstrated time and again that emotional competencies exert a positive influence on job performance and on interpersonal relations, as well as in coping with stress and promoting healthy living habits (Goleman, 1995; Extremera and Fernández-Berrocal, 2004; Molero et al., 2019). In psychology, these competencies are made operable with the concept of emotional intelligence, which was defined by Goleman as the ability of humans to come into contact with their own emotions, enabling them to respond adequately to different moods caused by internal or external agents (Goleman, 1995). Bar-On (1997) defined emotional intelligence as “A set of non-cognitive abilities, competencies, and skills that influence a person’s capacity for success by coping with environmental demands and pressures.” Based on this approach, he designed a questionnaire with which skills in each of the emotional competencies can be evaluated: *intrapersonal* (emotional understanding of oneself, assertiveness, self-concept, self-realization, and independence), *interpersonal* (empathy, social responsibility, and interpersonal relations), *adaptability* (reality test, flexibility, and problem-solving), *stress management* (tolerance to stress and impulse control), and *mood* (happiness and optimism) (Bar-On, 1996, 1997).

Based on the theoretical proposal of these authors, a multitude of studies have been undertaken to test the benefits of emotional intelligence. In adulthood, job adjustment and, in the healthcare professions in particular, emotional intelligence has been related to wellbeing, less stress, job satisfaction, and engagement (Brunetto et al., 2012; Görgens-Ekermans and Brand, 2012; Nel et al., 2013; Karimi et al., 2014; Zhu et al., 2015; Carvalho et al., 2018; Pérez-Fuentes et al., 2018).

Empathy is an emotional ability which enables one to connect with others. In general terms, empathy refers to one’s ability to put oneself in the place of others and read their state of mind, an ability neuropsychologically given by mirror neurons (Decety and Jackson, 2004; Sharmay-Tsoory et al., 2004; Rizzolatti and Sinigaglia, 2006). It involves interpreting and understanding what is happening to others, as well as personal identification with their emotions. It must be understood as a subjective phenomenon, because people interpret the reality of others based on their own experience, however, for healthy empathy with others, there can be no fusion between one’s own feelings and those of others (Fernández-Pinto et al., 2008). From this perspective, one’s interpretation of what others feel is more or less biased by one’s own experiences. Nevertheless, the experiences of another may also be interpreted correctly without emotionally connecting with them. Mindfulness studies have shown that its practice contributes to connecting with oneself, as it increases emotional self-awareness and facilitates the emotional connection with others, promoting transfer from a mental state to action, at the same time it improves emotional regulation, favoring emotional balance (Davis and Hayes, 2011; Amutio et al., 2018). Other studies have shown that mindfulness training, self-reflection, and social skills can help healthcare professionals recognize, regulate, and demonstrate empathy in clinical and professional contacts (Asuero et al., 2013; Oro et al., 2015; Verweij et al., 2018). Cognitive empathy (knowing what another feels) has been differentiated from emotional empathy (feeling what the other person feels) (Jolliffe and Farrington, 2006; Oliva et al., 2011; Merino-Soto and Grimaldo-Muchotrigo, 2015), although it is also argued that both types of empathy act together, and therefore, cannot be measured separately (Baron-Cohen and Wheelwright, 2004). From our perspective, we assume that there are different types of empathy and that they can be measured separately, following the theoretical proposal of Jolliffe and Farrington (2006). In healthcare professional teams, empathy facilitates teamwork and person-centered care (Lown et al., 2016; Orgambídez and de Almeida, 2017), and is related to subjective wellbeing (Pérez et al., 2019).

### Emotional Competency and the Healthcare Humanization Construct

The World Health Organization [WHO] (2015) defines humanization in nursing as a process of communication and mutual support between individuals, directed at transformation and understanding of the essential spirit of life. From the viewpoint of intervention in health, a recent systematic review on humanization-based intervention showed it to have substantial potential for increasing physical and emotional closeness between patient and healthcare professionals or between patients and their families (Galvin et al., 2018). From a psychological perspective, humanization refers to a style of interpersonal relations in which several psychological processes intervene, materialized in the acquisition of personal competencies (Pérez-Fuentes et al., 2019b). In the HUMAS model, the five essential personal competencies which define humanization in healthcare are (Pérez-Fuentes et al., 2019a): *Dispositional optimism*, which refers to positive expectations for the future; *sociability*, which is the ability to relate to others with assertiveness and empathy; emotional understanding, which involves understanding and interpreting properly the feelings of other persons; *self-efficacy*, which means confidence in acting appropriately to attain the expected results in potentially stressful situations; and *affection*, which consists of emotionally empathizing with the affective state of the other person without fusion with their feelings. From this perspective, humanization contributes to the integral development of the human being through a global approach to healthcare, where the patients become the center of the system and take on an active role along with the healthcare professional in caring for their own health. Studies specifically addressing the relationships between the humanization construct and emotional competencies are practically non-existent. Some findings of previous research on the variables above are discussed below.

Optimism generates positive expectations for the future and helps the individual to cope with stressful situations in professional practice (Mäkikangas et al., 2004; Segerstrom et al., 2017). It also improves psychological health, facilitates social relations (Seligman, 2006; Carver and Scheier, 2014), and prevents burnout (Vizoso and Arias, 2018). In self-efficacy, which is closely related to self-esteem (Pérez-Fuentes et al., 2019c), expectations depend on the emotional state (Bandura, 1997). Self-efficacy has also been considered a moderating variable of stress and offers protection against burnout and a better ability to cope with more problematic situations (Bodys-Cupak et al., 2016; Chang et al., 2016; Schönfeld et al., 2016; Shoji et al., 2016). Sociability involves relations based on empathy, assertiveness, and altruism (Bar-On, 2006; Bethlehem et al., 2017). Affection is a sense of maladjusted responsibility which can generate negative affect and diminish the quality of attention, and can even negatively influence the health of the healthcare professional (Pérez-Fuentes et al., 2018; Schwan, 2018). The role that positive affect has on positive mental health should also be considered. Insofar as it refers to emotional understanding, it is related to cognitive empathy, adopting the perspective, increasing awareness and reflexive capacity, and enabling emotions to be understood and managed effectively (Fernández-Berrocal and Pacheco, 2002; Fonagy and Bateman, 2007; Weiner and Auster, 2007; Decety and Fotopoulou, 2015; Howick et al., 2017; Pérez-Fuentes et al., 2019b). Other studies exploring the values of hospitality (such as respect, responsibility, quality, and transpersonal care) with nurses in Spain have highlighted its role in humanization of care and its connection with professional ethics (Bang et al., 2011; Arruda and Silva, 2012; Neves et al., 2013; Galán et al., 2017).

**FIGURE 1:**
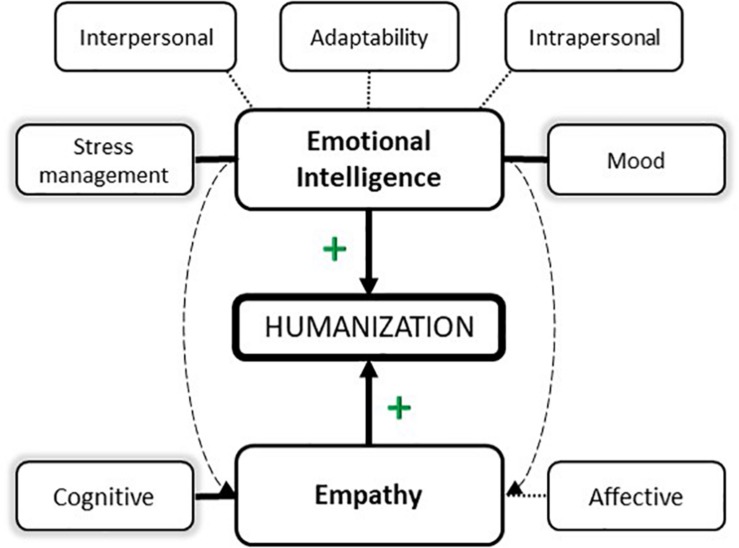
Hypothetical model proposal.

Based on the above theoretical proposals, and assuming that the work environment requires large amounts of emotional intelligence and empathy for humanization in healthcare, the following study was designed to analyze the role of both variables in the development of humanization in health. The main objectives were to: (1) Determine the explanatory value of emotional intelligence and cognitive empathy in humanization in a sample of nursing professionals and (2) explore the role of empathy in the relationship between emotional intelligence and humanization. With these objectives in mind, the following research hypotheses were proposed based on previous empirical evidence (Figure 1): H1: It was expected to discover significant positive correlations between emotional intelligence, empathy, and humanization in nursing professionals. H2: Certain components of emotional intelligence and empathy were expected to have a more explanatory weight in humanization. H3: Empathy was expected to play a mediating role between emotional intelligence and humanization.

## Materials and Methods

### Participants

A battery of questionnaires was answered by 338 nursing professionals. Cases with incongruent or random answers detected in a series of randomly distributed control questions were eliminated from the sample. This control system is based on questions with a single obviously correct answer, such as “*Right now I am answering a survey*.” Eight cases were found with wrong answers on the control questions. The final sample was therefore comprised of 330 Spanish nursing professionals with a mean age of 32.30 (SD = 7.54), in a range of 22–56. Participant distribution by gender was 83.9% (*n* = 277) women and 16.1% (*n* = 53) men, with a mean age of 32.62 (SD = 7.92) and 30.62 (SD = 4.90), respectively.

### Instruments

#### Healthcare Professional Humanization Scale (HUMAS; Pérez-Fuentes et al., 2019a)

This scale analyzes the professional’s humanization competencies focused on improving care. It consists of 19 items which measure professional competencies or attitudes: Dispositional optimism, Sociability, Emotional understanding, Self-efficacy, and Affection. The McDonald’s omega was calculated to estimate the reliability of each of the subscales: Dispositional optimism ω = 0.86, Sociability ω = 0.86, Emotional understanding ω = 0.88, Self-efficacy ω = 0.86, and Affection ω = 0.89. The omega for the complete scale was 0.88.

#### The Brief Emotional Intelligence Survey for Adults

*The Brief Emotional Intelligence Survey for Adults* (EQ-i-20M) adapted by Pérez-Fuentes et al. (2014) in an adult Spanish population was used. This version is made up of 20 items which measure five emotional intelligence components: Intrapersonal (ω = 0.87), Interpersonal (ω = 0.79), Stress management (ω = 0.82), Adaptability (ω = 0.83), and Mood (ω = 0.88).

#### Basic Empathy Scale (BES)

*Basic Empathy Scale* (BES) adapted by Merino-Soto and Grimaldo-Muchotrigo (2015), based on the brief form by Oliva et al. (2011), with Spanish adolescents of the original BES (Jolliffe and Farrington, 2006). It consists of nine items providing a score in Affective Empathy (feeling vicariously with another person), a score in Cognitive Empathy (which includes realizing what the other person feels), and a global empathy score. The reliability of the scales has an omega coefficient of 0.86 for Affective empathy and 0.90 for Cognitive empathy.

### Procedure

Before starting to collect the data, compliance with information standards, confidentiality, and ethics in data processing were guaranteed to the participants. The Bioethics Committee approved the study (Ref: UALBIO2019/30). The questionnaires were implemented on a Web platform which enabled them to be filled in online. Participants completed the tests voluntarily, with their express permission, anonymously and individually. For control of random or incongruent answers, a series of control questions were included, and any cases detected were discarded from the study sample.

### Data Analysis

This study was quantitative, observational, and cross-sectional. Correlational and descriptive analyses were carried out to identify the relationships between variables. The correlation analysis was based on Bayes factor inference on pairwise correlations for hypothesis comparison and estimation of the strength of evidence in favor of the alternative hypothesis over the null hypothesis. The descriptive statistics of the emotional intelligence components and empathy were also calculated by Humanization group (low, medium, high). An analysis of variance (ANOVA) was performed for intergroup comparison of means.

Identification of the possible Humanization predictors was done by stepwise multiple linear regression. Simple mediation analyses were also performed for the direct and indirect effects of the emotional intelligence and empathy variables on Humanization. The PROCESS macro for SPSS (Hayes, 2013) was used with bootstrapping with 5000 bootstrap samples. The Sobel test (Sobel, 1982; Kenny et al., 1998) was applied to compare the statistical significance of the direct and indirect effects through the mediator variable.

The McDonald (1999) coefficient omega was estimated to determine the reliability of the evaluation instruments used, following the recommendations of Ventura-León and Caycho (2017).

## Results

### Emotional Intelligence, Empathy, and Humanization: Correlations and Descriptive Statistics

Table 1 shows the Bayesian Pearson correlation matrix, where positive relationships may be observed between all the components of emotional intelligence and humanization. The cognitive component of empathy was correlated positively with humanization. The same was not true of the affective component of empathy, which had no significant relationship.

**TABLE 1:** Bayesian Pearson correlation pairs.

Pairs		Pearson’s *r*	BF10	95% CI	
				Lower	Upper
	INTRA	0.260***	6104.837	0.155	0.356
	INTER	0.492***	2.674e + 18	0.403	0.568
	STRESSM	0.349***	1.016e + 8	0.249	0.438
HUMAS	ADAPT	0.481***	3.204e + 17	0.392	0.559
	MOOD	0.618***	7.700e + 32	0.544	0.679
	AE	0.024	0.076	−0.084	0.131
	CE	0.432***	3.020e + 13	0.339	0.514

HUMAS = humanization, INTRA = intrapersonal, INTER = interpersonal, STRESS_M = stress management, ADAPT = adaptability, MOOD = mood, AE = affective empathy, CE = cognitive empathy. ***p < 0.001.

Moreover, in the Bayes factor inference on pairwise correlations, for the HUMAS ^[+]^ INTRA (Humanization ↔ Intrapersonal) pair, a BF_10_ showed that data were 6.104 × 10^3^ times more likely under H_1_ than H_0_, which provided extreme evidence in favor of a true correlation other than zero, and 95% confidence that the true correlation was between 0.15 and 0.35. In the comparison of the HUMAS ^[+]^ INTER (Humanization ↔ Interpersonal) pair, a BF_10_ found suggested that the data were 2.674 × 10^18^ times more likely under H_1_ than H_0_, providing extreme evidence in favor of a true correlation different from zero with a 95% confidence interval that the true correlation was between 0.40 and 0.50. For the HUMAS ^[+]^ STRESS_M (Humanization ↔ Stress management) pair, a BF_10_ showed that the data were 1.016 × 10^8^ times more likely under H_1_ than H_0_, providing extreme evidence in favor of H_1_, with 95% confidence that the true correlation was found between 0.25 and 0.40. In the HUMAS ^[+]^ ADAPT (Humanization ↔ Adaptability) pair, a BF_10_ indicated that the data were 3.204 × 10^17^ times more likely under H_1_ than H_0_, which provides extreme evidence in favor of H_1_, and 95% CI (0.39, 0.56). In the HUMAS ^[+]^ MOOD (Humanization ↔ Mood) pair, a BF_10_, showed that the data were 7.700 × 10^32^ times more likely under H_1_ than H_0_, which provides extreme evidence in favor of H_1_, and a 95% CI (0.54, 0.68).

In the humanization relationships with empathy, and specifically in the HUMAS ^[+]^ CE (Humanization ↔ Cognitive empathy) pair, BF_10_ = 3.020 × 10^13^, which provides extreme evidence in favor of H_1_ with a 95% CI (0.34, 0.51).

In addition, the mean scores on the emotional intelligence and empathy variables differed in the Humanization groups (low, mean, high) (Table 2). These differences were statistically significant in all cases except Affective empathy. Moreover, in the Bonferroni tests, differences were found between all the groups except in the Intrapersonal factor of emotional intelligence, where the differences were between the high and low Humanization levels.

**TABLE 2:** Emotional intelligence and empathy by humanization level.

HUM	Intrapersonal		
	*n*	M	SD
Low	101	10.38	2.48	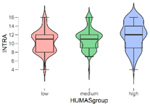
Medium	129	11.00	2.44	
High	100	11.77	3.05	
*F* = 6.83, *p* < 0.01				

	**Interpersonal**		
	***n***	**M**	**SD**

Low	101	11.49	2.08	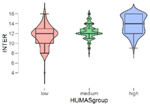
Medium	129	12.35	1.54	
High	100	13.74	1.86	
*F* = 38.89, *p* < 0.001				

	**Stress management**		
	***n***	**M**	**SD**

Low	101	12.40	2.53	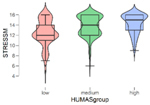
Medium	129	13.74	2.07	
High	100	14.25	1.91	
*F* = 19.39, *p* < 0.001				

	**Adaptability**		
	***n***	**M**	**SD**

Low	101	11.14	1.96	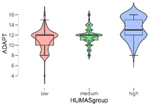
Medium	129	11.81	1.68	
High	100	13.31	2.16	
*F* = 33.45, *p* < 0.001				

	**Mood**		
	***n***	**M**	**SD**

Low	101	11.37	2.17	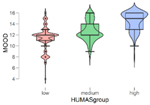
Medium	129	12.66	1.84	
High	100	14.47	1.72	
*F* = 65.90, *p* < 0.001				

	**Affective empathy**		
	***n***	**M**	**SD**

Low	101	13.50	2.73	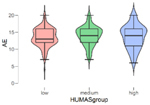
Medium	129	13.99	2.75	
High	100	13.66	3.44	
*F* = 0.81, *p* = 0.444				

	**Cognitive empathy**		
	***n***	**M**	**SD**

Low	101	18.25	2.99	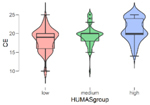
Medium	129	19.50	2.69	
High	100	21.07	2.98	
*F* = 24.14, *p* < 0.001				

*Descriptive statistics and boxplots. HUM = humanization, INTRA = intrapersonal, INTER = interpersonal, STRESSM = stress management, ADAPT = adaptability, MOOD = mood, AE = affective empathy, CE = cognitive empathy.*

In general, the highest mean scores in emotional intelligence and empathy (cognitive) were in the group with the highest Humanization levels.

### Emotional Intelligence and Empathy as Predictors of Humanization in Nursing

The regression analysis provided three models, of which the last had the most explanatory power with an explained variance of 51%. The factors included in the equation were Mood, Stress management, and Cognitive empathy (Table 3).

**TABLE 3:** Stepwise multiple linear regression model.

Humanization	Model	*R*	*R*^2^	*Corrected R^2^*	Change statistics					Durbin Watson
					Standard error of estimation	Change in *R*^2^	Change in *F*		Sig. change in *F*	
	1	0.61	0.38	0.38	6.49	0.38	202.57		0.000	1.71
	2	0.67	0.45	0.45	6.10	0.07	44.64		0.000	
	3	0.71	0.51	0.50	5.80	0.05	35.85		0.000	

	**Model 3**	**Unstandardized coefficients**		**Standardized coefficients**	***t***	**Sig.**	**95% CI**		**Collinearity**	
					
		***B***	**Std. error**	**Beta**			**Low**	**Upper**	**Tol.**	**VIF**

	(Constant)	28.68	2.83		10.11	0.000	23.11	34.26		
	MOOD	1.87	0.14	0.51	12.70	0.000	1.58	2.16	0.91	1.09
	STRESS_M	0.87	0.14	0.24	6.20	0.000	0.59	1.15	0.96	1.03
	CE	0.65	0.11	0.24	5.98	0.000	0.44	0.87	0.90	1.10

*MOOD = mood, STRESS_M = stress management, CE = cognitive empathy.*

Independence of residuals was analyzed to confirm model validity. The Durbin–Watson *D* was 1.71, confirming the absence of positive or negative autocorrelation. It was also observed that the *t* was associated with a probability of error below 0.05 in all cases. The standardized coefficients revealed that Mood was the factor showing the highest explanatory weight, followed by Stress management, and lastly, Cognitive empathy.

Finally, to check whether the relationship estimated was affected by multicollinearity, the Tolerance and Variance Inflation Factor (VIF) statistics were calculated. According to these values, absence of collinearity between the variables in the model may be assumed.

### Mediation Analysis of Cognitive Empathy in the Relationship Between Emotional Intelligence and Humanization

**FIGURE 2:**
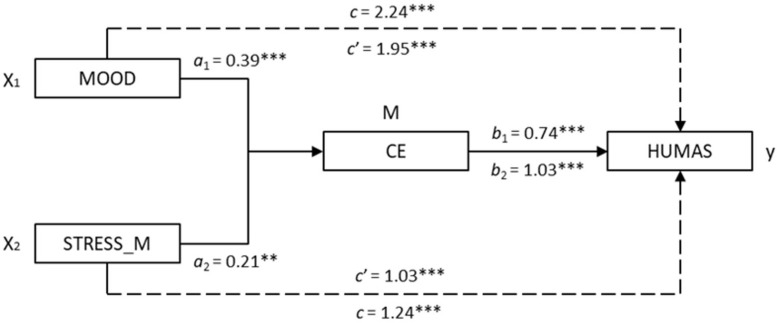
Mediation models of Cognitive empathy on the relationship between emotional intelligence (mood and stress management) and Humanization (Note. X_1_ = mood; X_2_ = stress management; M = Cognitive empathy; Y = Humanization. ^∗∗^*p* < 0.01, ^∗∗∗^*p* < 0.001).

Figure 2 shows the results of the simple mediation models, in which cognitive empathy was proposed as the mediator. In the first place, significant effects of the two components of emotional intelligence (X_1_, X_2_), MOOD (β = 0.39, 95% CI 0.25, 0.53), and STRESS_M (β = 0.21, 95% CI 0.06, 0.35) on cognitive empathy (M) were observed. The estimation of the total effect was significant in MOOD (β = 2.24, 95% CI 1.93, 2.55) and STRESS_M (β = 1.24, 95% CI 0.88, 1.61) in each of the models. Similarly, the direct effects on Humanization (X→Y) were significant in both the MOOD (β = 1.95, 95% CI 1.65, 2.26) and STRESS_M (β = 1.03, 95% CI 0.69, 1.36) predictors.

Finally, the analysis of indirect effects (X→M→Y) with bootstrapping found significant values in both models computed: MOOD → CE → HUMAS [β = 0.29, SE = 0.08, 95% CI (0.16, 0.48)] and STRESS_M → CE → HUMAS [β = 0.21, SE = 0.08, 95% CI (0.06, 0.42)]. The results of the Sobel test, which reflects the effect size of mediation described in the models, were statistically significant with *Z* = 4.14, *p* < 0.001, y *Z* = 2.70, *p* < 0.01, respectively.

## Discussion

The empirical study presented above met both objectives originally posed, to determine the explanatory value of emotional intelligence and empathy with respect to professional humanization competencies in a sample of nursing professionals, and to explore the mediating role of empathy in relation to emotional intelligence and humanization competencies.

In the first place, the results showed that all the components of emotional intelligence correlated positively with humanization of professionals, with a large effect size in all cases, except the Intrapersonal component of emotional intelligence, where the effect size was only medium. These findings coincide with our first research hypothesis in which it was expected *a priori* to find positive relationships between emotional intelligence and humanization. In the bibliography reviewed, we found results that support our findings. Thus, emotional intelligence has been related with engagement (Brunetto et al., 2012; Zhu et al., 2015; Pérez-Fuentes et al., 2018) and job satisfaction (Görgens-Ekermans and Brand, 2012; Nel et al., 2013; Karimi et al., 2014; Carvalho et al., 2018). These conditions can favor humanized care and improve care quality.

Similarly, the results of our research showed that only the cognitive component of empathy was significantly and positively correlated with humanization, thereby confirming our hypothesis. Cognitive empathy must be understood as the process of putting oneself mentally in the place of the other, thereby adopting their perspective. The cognitive component of empathy facilitates the recognition and regulation of emotions (Pérez-Fuentes et al., 2019a). Our data in this respect showed that high scores in cognitive empathy were associated with high scores in humanization, while affective empathy was not significantly correlated with it. Other studies have shown that high affective empathy can lead to feelings of excessive responsibility, and have a negative effect on one’s perception of self-efficacy and self-esteem (Schwan, 2018; Pérez-Fuentes et al., 2019c). Therefore, the levels of affective empathy (feeling what others feel) and of affection in humanization of care should be kept at moderate levels.

Second, it was found that emotional intelligence and empathy explained 51% of the variability in humanization, where the mood and stress management components and Cognitive empathy were the strongest predictors of humanization in nursing, in that order. These empirical data offer information about various components of emotional intelligence and empathy having different weights in the explanation of humanization, and emphasizing the fundamental role of these three variables (Cognitive empathy, Mood, and Stress management), which explained over 50% of the variability found in humanization in nursing.

Finally, as we advanced in the study, our objective of analyzing the mediating role of empathy in the relationship between emotional intelligence and humanization, where cognitive empathy acts as the mediator variable in the relationship between the Mood component and Humanization, and the Stress management component and Humanization, was also met. The findings showed that Mood exerted an effect on cognitive empathy and this, in turn, on humanization. Stress management also had an effect on cognitive empathy, which then had an effect on humanization. These mediation models emphasize the mediating role of cognitive empathy in stress management and improvement in mood and its relationship with humanization. Thus, the study could be widened to analyze the role of positive affect and its relationship with optimism for coping with negative mood or stressful situations at work, and also, the role of dispositional factors linked to affective empathy with regard to the level of activation or arousal and the emotional intensity with which different situations are experienced.

This study had some limitations in both the study design, which was cross-sectional, and therefore no causal relationships could be established between variables, and the self-report measures used to evaluate them (humanization, emotional intelligence, and empathy). While they are very useful for studies with large samples, for their low-cost application and data processing, their use was limited here. However, this study could be replicated with larger samples to broaden and clarify the practical applications derived from the variables analyzed and their relationship to humanization, bearing in mind the cultural and psychological differences in the nurses working in different healthcare sectors. To our knowledge, our study is the first to explore the relation between humanization, empathy, and emotions management as key to reducing the burnout and to achieving a positive work environment for nurses.

When nurses suffer any unpleasant experiences with patients or other healthcare professionals, nurse staff expect nurse managers’ support. If this is not given, then nurses could consider this like an organization betrayal, which increases burnout, job dissatisfaction, and even absenteeism (Brewer et al., 2019). However, nurse managers’ and nurse leader’s empathy and humane treatment are relevant in the nursing context. This provides the nursing staff with a way to improve their self-esteem, formation about emotions and stress management, or interventions to reduce burnout. It could also promote organizational behaviors and healthy work environments in clinical settings, foster more job-related learning, and even improve the quality of care (Mortier et al., 2015; Wilkinson et al., 2017; Feather et al., 2018).

## Conclusion

This study is a pioneer in research on the psychological perspective of the humanization of care by healthcare professionals. The results show that mood and stress management—both components of emotional intelligence—and cognitive empathy explain over half of the variability found in the humanization of care competencies in a sample of nurses in the Spanish context. It further proposes two mediation models in which cognitive empathy acts as the intermediary in the relationship between stress management and humanization and between mood and humanization.

These findings emphasize the role of emotional competencies in the quality of patient care from an approach of personal competencies in the humanization of healthcare. Its practical implications would enable the development of psychological competencies and tools for healthcare professionals that reinforce the expression of feelings and emotional regulation so they can cope successfully with potentially stressful situations in clinical practice.

Professionals should therefore have spaces for both reflection and for training that facilitate the acquisition of competencies, skills, and attitudes providing nurses, in this case, with the tools for practicing their profession through the prism of humanization and diminish their risk of burnout. It would also be advisable to introduce humanization of care competencies in both university and non-university training of healthcare professionals.

## Data Availability Statement

The datasets generated for this study are available on request to the corresponding author.

## Ethics Statement

The studies involving human participants were reviewed and approved by the Bioethics Committee of the University of Almería (Ref: UALBIO2019/30). Written informed consent to participate in this study was provided by the participants.

## Author Contributions

MP-F contributed to the concept, design, analysis, and interpretation of the data. IH-P contributed to the concept, design, and interpretation of the data. MM contributed to the concept, design, analysis, and interpretation of the data. NO contributed to the technical details and manuscript preparation. DA-M contributed to collecting the data. JG contributed to critically revising the manuscript for important intellectual content and the final approval of the version to be published. All authors accepted and agreed that the work is original; any methods and data presented are described accurately and honestly; and any relevant interests have been disclosed.

## Conflict of Interest

The authors declare that the research was conducted in the absence of any commercial or financial relationships that could be construed as a potential conflict of interest.
